# How Is the Self-Perceived Work Ability Affected by the Duration of Unemployment, Heart Rate Variability and the Amount of Physical Activity in Older Long-Term Unemployed Persons?

**DOI:** 10.3390/ijerph17062039

**Published:** 2020-03-19

**Authors:** Anke Bumann, Daniel Niederer, Constanze Santarossa, Winfried Banzer, Lutz Vogt

**Affiliations:** 1 Department of Sports Therapy and Exercise Physiology, Justus-Liebig-University Giessen, 35390 Giessen, Germany; Anke.Bumann@sport.uni-giessen.de; 2 Department of Sports Medicine and Exercise Physiology, Goethe University Frankfurt, 60487 Frankfurt am Main, Germany; constanze.santarossa@gmail.com (C.S.); l.vogt@sport.uni-frankfurt.de (L.V.); 3 Institute for Occupational Medicine, Social Medicine and Environmental Medicine, University Hospital Frankfurt, 60596 Frankfurt am Main, Germany; banzer@med.uni-frankfurt.de

**Keywords:** quality of life, activities of daily life, healthcare workers, prevention

## Abstract

Background: This study investigated whether work ability is associated with the duration of unemployment, heart rate variability (HRV), and the level of physical activity. Methods: Thirty-four unemployed persons (mean 55.7 ± standard deviation 33.3 years, 22 female, 12 male, unemployed: range 1–22.5 years) participated in the cross-sectional study. The Work Ability Index (WAI) and International Physical Activity Questionnaire (IPAQ) were applied. Short-term (five minutes) resting HRV (Low Frequency (LF), High Frequency (HF), Total Power (TP)) was collected. Results: Work ability was positively associated with the HRV: LF (r = 0.383; *p* = 0.025), HF (r = 0.412; *p* = 0.015) and TP (r = 0.361; *p* = 0.036). The WAI showed a positive linear correlation with the amount of total physical activity (r = 0.461; *p* = 0.006) as well as with the amount of moderate to vigorous physical activity (r = 0.413; *p* = 0.015). No association between the WAI and the duration of unemployment occurred. Conclusions: the relation between self-perceived work ability, health-associated parameters, the HRV and the level of physical activity points out the relevance of health-care exercise and the need of stress-reducing interventions to improve perceived work ability. Our results point out the need for the further and more holistic development of healthcare for the unemployed.

## 1. Introduction

There is a close relationship between unemployment and health [1]. In particular, unemployment may become a psychosocial stressor [2] with long-term consequences on work ability and the overall state of health [3]. Heart rate variability (HRV) is a global measure of the activity of the autonomic nervous system [4] which mirrors stress levels, their pathological effects, and the body’s ability to tolerate stress situations [5]. In detail, the HRV and its different subscales mirror the sympathetic as well as parasympathetic activities of the autonomic nervous system (ANS) [6]. Lower HRV values are associated with a better ability to tolerate stress situations. Initial studies focusing on HRV measurements in unemployment revealed that the unemployed had a decreased HRV in comparison to the employed [7].

Hollederer [8] underlined a cycle of health impairments in the unemployed leading to decreased re-employment opportunities. Unemployment may thus lead to both a decrease in work ability and stress/health status. The latter two may additionally fuel the duration of the unemployment in a vicious cycle. Several studies further observed a decrease in the amount of physical activity in unemployed compared to employed persons [9,10] This lack of health-enhancing physical activity may further worsen the vicious cycle. In such a vicious cycle of reduced work ability, increased stress levels, and lower physical activity exists, specific stress-reducing work-ability-enhancing or physical-activity-promoting programs may be able to counteract against this process.

However, it is still unknown if the impairments described are linked to work ability, which would be a first hint for the hypothesised vicious cycle, or be rated as independent of one another. In this context, the purpose of the present study was to investigate whether self-perceived work ability is associated with the duration of unemployment, HRV and the amount of physical activity in older, long-term unemployed persons.

## 2. Materials and Methods

### 2.1. Study Type and Ethical Aspects

The present study adopted a cross-sectional design. All participants subscribed informed consent prior to study participation. The study was previously approved by the local Ethics Committee (approval number 2017–29). The study was planned and performed in agreement with the Declaration of Helsinki (Version Fortaleza 2013).

### 2.2. Participants

We enrolled 34 participants (22 women, 12 men, unemployed > 1 year) aged 51 to 64 years. The main criterion for inclusion was the lack of pre-participation in a health-related intervention for the unemployed. The participants were clients of a job centre at the time of measurement and were recruited during the consultation hours for the older unemployed persons (>50 years) by independent researchers (not by job centre representatives). Exclusion criteria were chronic infection, uncontrolled hypertension (diastolic pressure over 95 mmHg) and medical conditions limiting participation in any exercise testing.

### 2.3. Experimental Design and Outcome Assessment

All outcomes were assessed in a standardised order and following demographic measurements: self-perceived work ability, amount of physical activity and HRV parameters. The sociodemographic variables assessed were: sex, age (years), body mass index BMI (kg/m²), professional qualification, nationality, and the duration of unemployment (years). The duration of unemployment was ascertained by means of a single open question which had to be confirmed by a job centre representative.

### 2.4. Work Ability Index

The Work Ability Index (WAI) assesses the self-perceived work ability [11]. The WAI is a questionnaire which can be used in various settings and has been assessed in numerous clinical studies as a suitable ’early warning instrument’ for early retirement and mortality [12]. For the current study, one question was used: the current self-perceived work ability rated with a 10-scaled numerical rating scale (NRS; 0 = incapacity for work, 10 = best work ability ever achieved).

### 2.5. Heart Rate Variability

The HRV was measured in accordance with the guidelines of the Task Force of the European Society of Cardiology [13]. Due to spatial configuration, the HRV was recorded while sitting. The position has, for short-term resting HRV, no impact on the results [14]. The procedure included a rest of ten minutes in a sitting position, measuring the heart rate (R-R-intervals) using the Polar V800 (Polar Electro GmbH, Deutschland). Since a measurement by electrocardiogram ECG was not possible for organizational reasons, the V800 was an appropriate and valid alternative [15] for monitoring HRV. A 5-min interval with a stable heart rate was selected for analysis. The following frequency-based HRV parameters were calculated (Kubios software version 2.2; University of Eastern Finland [16]) using a fast Fourier transformation (FFT): Low Frequency (LF; 0.04–0.15 Hz), High Frequency (HF; 0.15–0.4 Hz), and Total Power (TP). In order to assure a normal distribution, all HRV parameters were further analysed using their logarithmised (ln) values.

### 2.6. International Physical Activity Questionnaire

To monitor the physical activity, the International Physical Activity Questionnaire (IPAQ) for the self-assessment of movement behaviour was used. The questionnaire is used for self-assessment of movement behaviour and shows moderate reliability in test-retest design with regard to repeatability [17]. This seven-item-questionnaire records the amount of physical activity in the last seven days. Low, moderate and vigorous intensity amounts (days/week, hours and minutes per day) as well as the daily sitting time were assessed. From these values, the total amount of physical activity (pA) per week and the total amount of moderate to vigorous physical activity (MVPA) were calculated.

### 2.7. Statistical Analysis

Statistical processing was carried out using Bias for Windows (version 11.0). For the descriptive analysis, the means and, median, the minimum to maximum ranges, and standard deviation were examined. The test for the normal distribution of residuals was carried out using the Kolmogoroff–Smirnoff–Lilliefors test. Simple linear correlation analyses according to Pearson using product–moment correlation coefficients were performed for the detection of potential associations between the dependent variable WAI and the independent variables duration of unemployment, HRV, natural logarithm of the Low Frequency (lnLF), natural logarithm of the High Frequency (lnHF), natural logarithm of the Total Power (lnTP)) and the IPAQ-values pA and MVPA. The significance level was fixed at *p* < 0.05.

## 3. Results

### Participants’ Characteristics

No participant withdrew his/her consent for participation. Due to incomplete datasets, one participant had to be excluded. The participants’ demographics, their educational status and unemployment characteristics are displayed in Table 1.

## 4. Main Results

**Figure 1:**
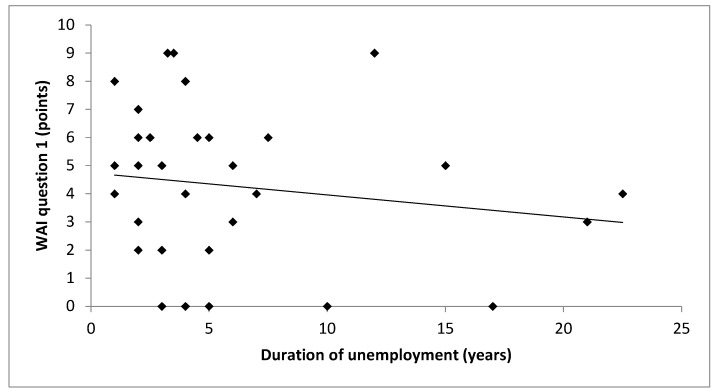
Scatter diagram between duration of unemployment and self-perceived work ability (WAI question 1) including a regression line. WAI: work ability index.

**Figure 2:**
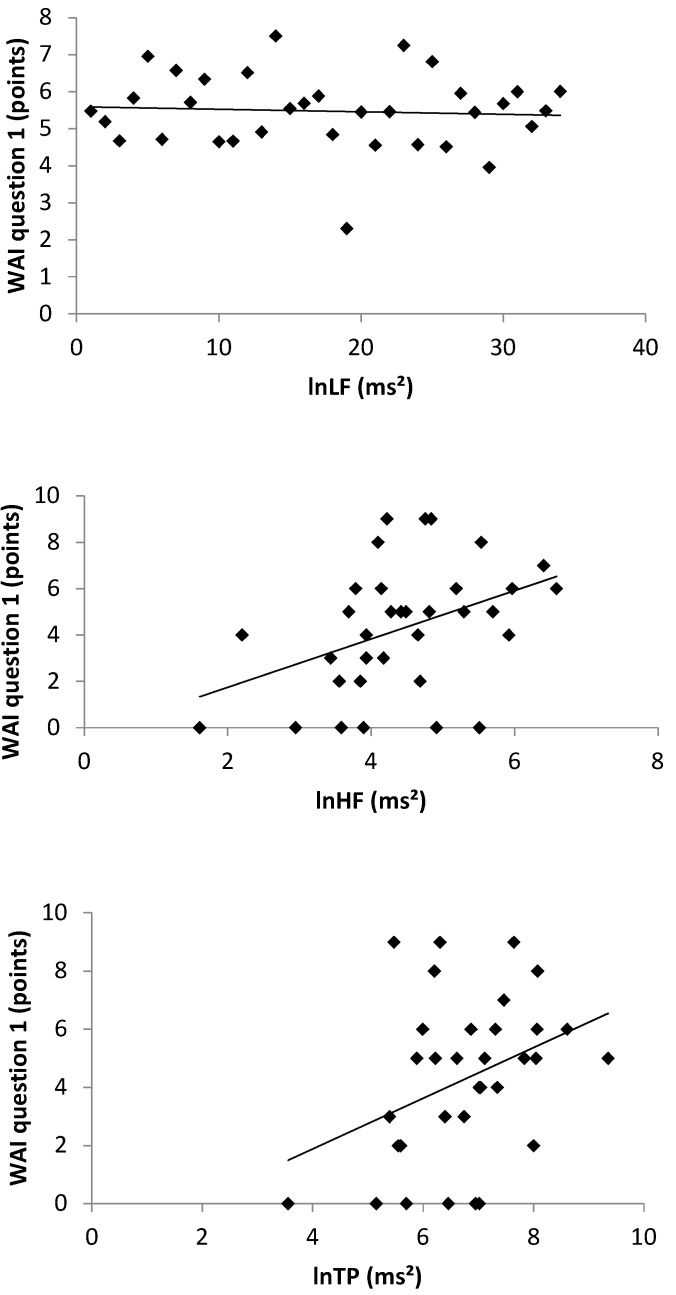
Scatter diagrams between the self-perceived work ability (WAI question 1), lnLF, lnHF and lnTP with the corresponding regression lines. WAI: work ability index, lnLF: natural logarithm of the Low Frequency, lnHF: natural logarithm of the High Frequency, lnTP: natural logarithm of the Total Power.

**Figure 3:**
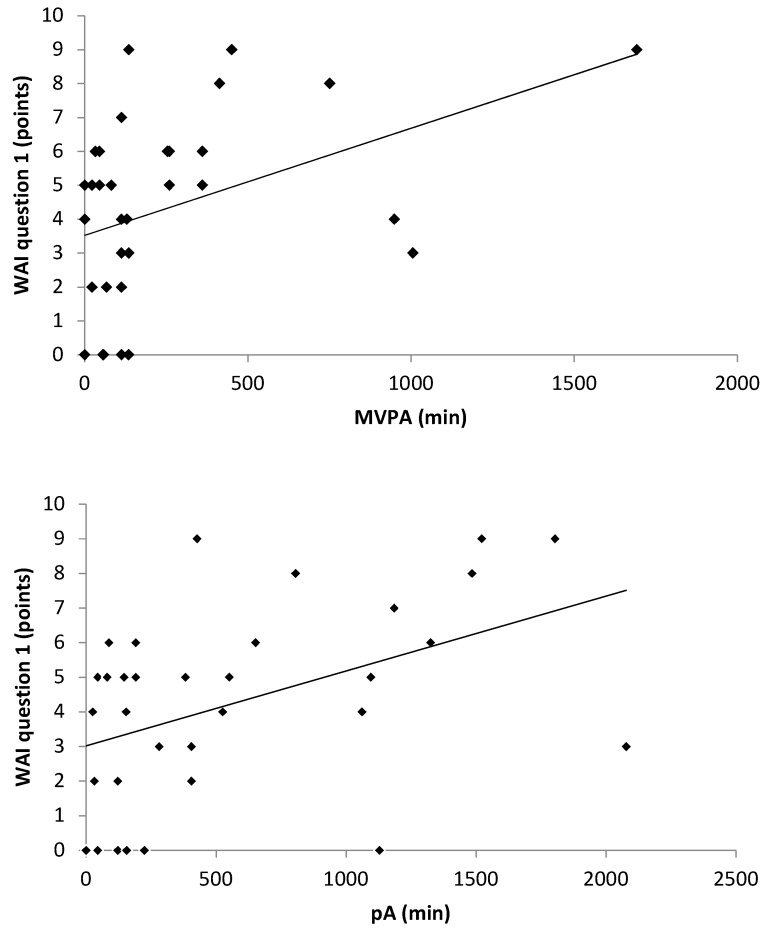
Scatter diagrams between self-perceived work ability (WAI question 1), physical activity (pA) and MVPA of the past seven days with regression lines. WAI work ability index, pA physical activity, MVPA moderate to vigorous physical activity.

Between the WAI and the duration of unemployment, no systematic linear correlation occurred (Figure 1) (r = 0.157, *p* = 0.375). As Figure 2 illustrates, positive linear correlations were found between the WAI and all HRV parameters: LF (r = 0.383; *p* = 0.025), HF (r = 0.412; *p* = 0.015) and TP (r = 0.361; *p* = 0.036). The WAI and the amount of total physical activity (r = 0.461; *p* = 0.006) as well as the moderate to vigorous physical activity (r = 0.413; *p* = 0.015) showed a positive linear correlation, as depicted in Figure 3.

## 5. Discussion

We found that HRV and physical activity, but not the duration of unemployment, are related to self-perceived work ability. The higher the work ability, the lower the HRV values, and the lower the stress level, the more physically active the participants were.

The work ability, all HRV values and the amount of physical activity of unemployed persons are lower/worse than those of employed persons [18]. This can be confirmed by our results, and the values of Jandackova et al. [18] and ours are comparable.

### 5.1. Circumstances of Unemployment as an Psycho-Social Stressor

All HRV measurements (LF, HF and TP) of the participants in the current study were lower than those of the reference working population [13]. The data presented in this paper are therefore considered representative of the underlying population. Sustainable and stress-related unhealthy attitudes enhance the risk of poor work ability [19]. Simultaneously, poor self-assessed work ability predicts future long-term sickness absence, disability pension and long-term unemployment [20]. Beyond these general associations, Worach-Kardas and Kostrzewski [21] highlighted a correlation between unemployment and chronic stress. Possible reasons for the association (and suggestions for a causal relationship) between HRV and self-perceived work ability must be discussed in the context of unemployment as stress triggering [2].

Low HRV values, as present in the current participants, can be caused by stress of any kind (psychological, physical, cellular or psychosocial) [22]: if the organism is exposed to a chronic stressful situation, the sympathetic tone prevails over a longer time period, the activity of the parasympathetic nervous system is lower and an imbalance between these two parts of the ANS develops [23]. As a result, the function of the parasympathetic nervous system, which is used for the regeneration of the organism, does not further appropriately achieve its objective. Consequences may be psychological impairments but also organic dysfunctions [2]. As stated above, unemployment, in particular long-term unemployment, can be such a chronic stressor situation and negative affective component [24]. Psychosocial aspects such as social participation or self-esteem, material factors and capital can be impaired by long-term unemployment. The results of our study, therefore, support the implementation of stress-reduction measures, as proposed in [25] as well.

### 5.2. The Longer Unemployed, the Lower the Work Ability?

The results further reveal that the duration of unemployment within the current study population is not related to self-perceived work ability. In contrast, several studies have shown that there might be an association between long-term unemployment and work ability. The results of Lange and Lampert [26] showed a significantly reduced earning capacity of long-term unemployed men. As Worach-Kardas and Kostrzewski [21] highlighted in their study, long-term unemployed persons rated their quality of life, physical and mental health as well as social components worse than those who were short-term unemployed. Beyond these group differences, no linear association was found.

As Lundin et al. [20] underlined, self-assessed work ability turns out to be an important factor and indicator for future labour market exclusions and public health monitoring. As an interrelationship between the parameters work ability, HRV, and the amount of physical activity is given and may be causal, improving one of the parameters may facilitate the others. The association between the WAI and the amount of physical activity found in our study supports the potential relevance of exercise as a health resource in this population. This knowledge could be relevant to strengthen self-perceived work ability through movement interventions.

### 5.3. Benefits of Physical Activity

Various studies have further shown that physical activity can have a positive effect on HRV: Soares-Mirnada [27] pointed out positive indices of autonomic function in older adults due to habitual physical activity and concluded a lower cardiovascular mortality. Exercise and physical activity thus may be a relevant intervention in such populations, also due to the positive effects of exercise on the depressive symptoms of the unemployed [28]. One proven intervention approach is exercise in combination with exercise counselling [29]. A higher level of physical activity seems to be related to a better work ability and physical activity can be used as a predictive tool for the potential deterioration of work ability [30,31]. These results strengthen the idea of promoting and facilitating the engagement of employees in physical activity and the need for maintaining a physically active lifestyle [32,33].

### 5.4. Limitations

In the current study, we assessed work ability using a 10-point Likert scale but with a single question only. A more detailed assessment may have revealed even more meaningful values. Moreover, physical activity was recorded with the IPAQ, which tends to overestimate the amount of physical activity [34] but shows a good practicability. For further studies, this method of measuring physical activity could be replaced by methods such as accelerometery [35]. For further investigations concerning the potential correlations between HRV and physical activity, a follow-up study design with exercise interventions should be performed, as well as with a higher number of test persons.

### 5.5. Implications for Practice

Many health promotion projects already use the positive effects of regular physical activity to improve well-being and quality of life and other research proposes that the positive effects of exercise and a strong social background could be conducive for good health in the unemployed. However, the consideration of all aspects, physiological (HRV and physical activity) as well as mental resources (self-perceived work ability), is essential to strengthen health in the unemployed.

## 6. Conclusions

An association of work ability, HRV and the amount of physical activity is evident in the long-term unemployed.

Our results point out the need for the further and more holistic development of healthcare for the unemployed. The potential to influence work ability and HRV through exercise and exercise counselling may not be neglected in the future, which still has to be delineated in a future study.

## Figures and Tables

**Table 1:** Participants’ anthropometrics, demographics, education and duration of unemployment. If not stated otherwise, the data are displayed as the mean ± standard deviation; *n* (number); WAI (Work Ability Index); HRV (heart rate variability); lnLF (natural logarithm of the Low Frequency); lnHF (natural logarithm of the High Frequency); lnTP (natural logarithm of the Total Power); IPAQ (International Physical Activity Questionnaire); MVPA (moderate to vigorous physical activity).

Sex (*n*)	Female: 22	Male: 12
Age (Year)	55.71 ± 3.31	
BMI (kg/m^2^)	31.73 ± 6.55	
**Professional Qualification**	***n***	**%**
none	9	26
yes	21	62
university degree	3	9
unknown	1	3
**Nationality**	***n***	**%**
German	25	74
Other	9	26
**WAI Question 1 (point) 0–10**	4.29 ± 2.76	
**Duration of Unemployment (years)**	***n***	**%**
1–5	24	71
6–10	5	15
11–15	3	9
16–20	1	3
21–25	1	3
total	5.76 ± 5.52	
**HRV (ms^2^)**		
lnLF	5.48 ± 1.02	
lnHF	4.44 ± 1.09	
lnTP	6.77 ± 1.14	
**Physical Activity (IPAQ) (min)**		
total physical activity	2077.5 ± 393.75	
moderate to vigorous physical activity MVPA	244.63 ± 360.47	
